# Knock-Down of GPR88 in the Dorsal Striatum Alters the Response of Medium Spiny Neurons to the Loss of Dopamine Input and L-3-4-Dyhydroxyphenylalanine

**DOI:** 10.3389/fphar.2019.01233

**Published:** 2019-10-25

**Authors:** Manuela Ingallinesi, Benjamin Galet, Jonathan Pegon, Nicole Faucon Biguet, Anh Do Thi, Mark J Millan, Clotilde Mannoury la Cour, Rolando Meloni

**Affiliations:** ^1^Department of Biotechnology and Biotherapy, Institut du Cerveau et de la Moelle épinière (ICM) UPMC/INSERM U 1127/ CNRS UMR 7225, CHU Pitié-Salpêtrière, Paris, France; ^2^Center for Innovation in Neuropsychiatry, Institut de Recherches Servier, Croissy sur Seine, France

**Keywords:** Gpr88, 6-hydroxydopamine 6-OHDA, turning behavior, lentiviral vector, L-3-4-dyhydroxyphenylalanine–induced dyskinesia

## Abstract

The effects of L-3-4-dyhydroxyphenylalanine (L-DOPA) treatment for replacing the dopamine (DA) loss in Parkinson’s disease (PD) progressively wear off and are hindered by the development of dyskinesia, prompting the search for new treatments. The orphan G protein-coupled receptor 88 (Gpr88) represents a potential new target, as it is highly and almost exclusively expressed in the projecting gamma-Aminobutyric Acid-ergic (GABAergic) medium spiny neurons of the striatum, is implicated in motor activity, and is downregulated by 6-hydroxydopamine (6-OHDA) lesions, an effect that is reversed by L-DOPA. Thus, to evaluate Gpr88 as a potential target for the management of PD and L-DOPA–induced dyskinesia (LID), we inactivated Gpr88 by lentiviral-mediated knock-down with a specifically designed microRNA (miR) (KD-Gpr88) in a 6-OHDA rat model of hemiparkinsonism. Then, we investigated the effects of the KD-Gpr88 in the DA-deprived dorsal striatum on circling behavior and LID as well as on specific markers of striatal neuron activity. The KD-Gpr88 reduced the acute amphetamine-induced and increased L-DOPA–induced turning behavior. Moreover, it normalized the upregulated expression of striatal *Gad67* and *proenkephalin* provoked by the 6-OHDA lesion. Finally, despite promoting ΔFosB accumulation, the KD-Gpr88 was associated neither with the upregulation of *prodynorphin*, which is causally linked to the severity of LID, nor with the aggravation of LID following chronic L-DOPA treatment in 6-OHDA–lesioned rats. These results thus justify further evaluation of Gpr88 as a potentially novel target for the management of PD as an alternative to L-DOPA therapy.

## Introduction

The loss of dopamine (DA) input to the GABAergic striatal medium spiny neurons (MSNs) provokes an imbalance between the direct stimulatory striato-nigral and the indirect inhibitory striato-pallidal pathways responsible for movement initiation, thereby inducing the motor symptoms of Parkinson’s disease (PD) (Gerfen and Surmeier, 2011). The altered activation of these striatal outputs is associated with a net increase of the striatal glutamic acid decarboxylase 67 (*Gad67*) expression and the concomitant downregulation of prodynorphin (*Pdyn*) in the DA type 1 (D1) receptor regulated striato-nigral direct pathway as well as upregulation of proenkephalin (*Penk*) in the DA type 2 (D2) receptor regulated striato-pallidal indirect pathway (Carta et al., 2001). L-DOPA replacement therapy alleviates the motor symptoms but in the long term, by hyperactivating the sensitized D1 receptor in the direct stimulatory pathway, provokes L-DOPA–induced dyskinesia (LID) (Spigolon and Fisone, 2018) by a complex pattern of interaction with cellular substrates and neural networks (De Deurwaerdère et al., 2017) including the accumulation of ΔFosB (Nestler, 2015), which upregulates *Pdyn* in the MSN of the direct pathway (Andersson et al., 1999; Andersson et al., 2003). Thus, alternative antiparkinsonian targets, avoiding—or masking—the untoward effects of L-DOPA therapy and offering new therapeutic approaches, are needed for the treatment of PD (Huot et al., 2013; Fox et al., 2018).

Gpr88, an orphan G protein–coupled receptor almost exclusively expressed in the striatum (Mizushima et al., 2000), specifically in the MSN (Massart et al., 2009), displays several features of a potential target for the treatment of PD. Namely, Gpr88 knock-out (KO) mice display DA hypersensitivity, suggesting that Gpr88 may have an inhibitory influence on DA-dependent MSN activity (Logue et al., 2009; Quintana et al., 2012). Reciprocally, DA may modulate Gpr88 activity, since DA loss following 6-hydroxydopamine (6-OHDA) lesions of the DAergic nigrostriatal pathway downregulates *Gpr88* expression, which is thereafter restored by L-DOPA (Massart et al., 2009; Massart et al., 2012). However, the interplay between DA signaling and Gpr88 activity is not straightforward, since in basal conditions, the levels of Gpr88 expression are twofold higher in the MSN of the indirect pathway as compared to the MSN of the direct pathway (Massart et al., 2009). Moreover, the Gpr88 downregulation associated with DA loss is the net result of an increase of Gpr88 expression in the direct pathway and a decrease of its expression in the indirect pathway, hinting that Gpr88 responds differently to specific D1 or D2 receptor stimulation, while the L-DOPA treatment completely reverses the 6-OHDA–induced alterations of Gpr88 expression in both pathways (Massart et al., 2009). Finally, while the Gpr88 KO results in increased locomotion in response to DA stimulation (Logue et al., 2009), we have previously shown that the local inactivation of Gpr88 in the ventral striatum decreases the motor hyperactivity induced by amphetamine (Amph) (Ingallinesi et al., 2015). Thus, the precise role played by Gpr88 in motor regulation remains unclear, and its relevance as a target for PD treatment needs to be further evaluated.

To this end, using the unilateral 6-OHDA lesion as a model of PD, we locally inactivated Gpr88 in the dorsal DA-deprived striatum, a region that is associated with motor regulation (Do et al., 2013). We then assessed the impact of the Gpr88 knock-down (KD-Gpr88) on the turning behavior induced by Amph and L-DOPA, on the development of LID, and on the expression of striatal markers altered by the 6-OHDA lesion and the chronic L-DOPA treatment such as *Gad67* as a marker of general MSN activity (Cenci et al., 1998) as well as *Pdyn* and *Penk* as markers of direct and indirect pathway specific activity, respectively (Cenci et al., 1998; Steiner and Gerfen, 1998).

## Materials and Methods

### Experimental Animals

Six-week-old male Sprague Dawley rats (Janvier Labs, Rte des chênes secs, 53940 Le Genest-Saint-Isle, France) weighing 250–270 g at the beginning of the experiments were housed on a 12 h dark/light cycle at 20–22°C with free access to food and water. Animal studies authorized by “Ministère de la Recherche” (APAFIS #3669-2016011817516297 v6) were conducted in an approved animal facility (agreement #B75-13-19). The animals were handled throughout the study in compliance with the European Union 2010 Animal Welfare Act and the 2010/63 French directive.

### Drugs

6-Hydroxydopamine hydrobromide (6-OHDA), d-amphetamine sulfate (Amph), L-3,4-dihydroxyphenilalanine methyl ester hydrochloride (L-DOPA), and benserazide were purchased from Sigma-Aldrich. 6-OHDA was freshly prepared in physiological saline solution plus 0.02% ascorbic acid. The other drugs were freshly prepared in physiological saline solution only.

### Lentiviral Vectors

Lentiviral (LV)-vectors (p24 of stock solutions ranging between 200 and 300 ng/µl and transducing units/ml around 2 × 10^8^) co-expressing, under the drive of the ubiquitous phosphoglycerate kinase (PGK) promoter, the Emerald green fluorescent protein (EmGFP) and a designed microRNA (miR), either specifically directed against the Gpr88 mRNA (LV-miR-Gpr88) or without any target in the rat genome (LV-miR-control), were produced according to standard procedures (Zennou et al., 2001; Castaing et al., 2005).

### Experimental Design

**Figure 1:**
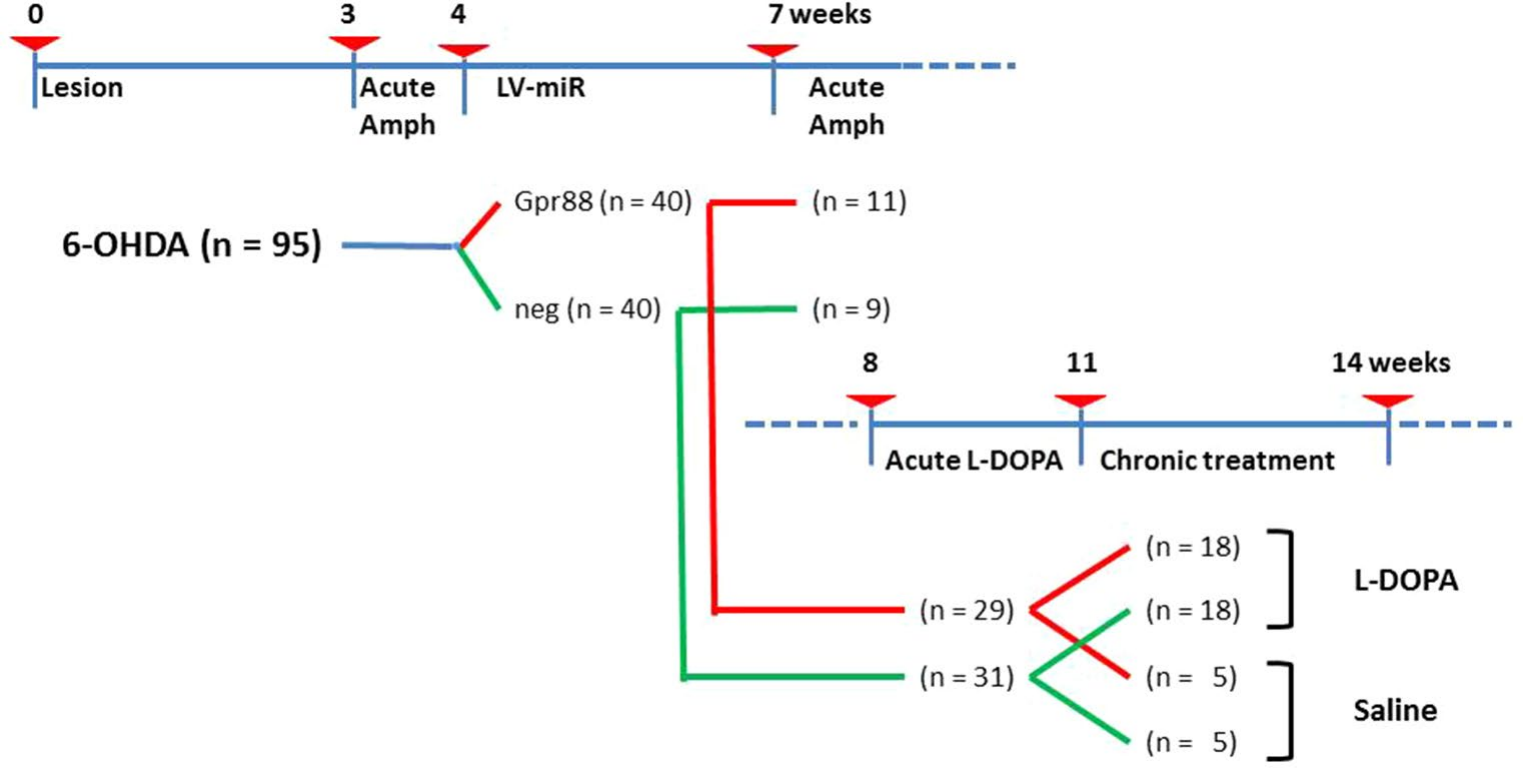
Experimental design. Timeline in weeks showing the different experimental settings and animal groups. Number of animals used in each group is reported in parentheses.

The experimental design including the timeline, the different steps, and the number of animals involved is presented in **Figure 1**. Briefly, 6-week-old rats (n = 95) at the starting time (T-0) received stereotaxic 6-OHDA injections. Three weeks later (T-3), these rats were tested for acute Amph-induced turning behavior. The rats not showing individual means > 5 full turns per minute in the direction ipsilateral to the lesion were discarded (n = 15). At T-4, the remaining 6-OHDA–lesioned rats (n = 80) received stereotaxic injections of lentiviral vectors expressing either the designed miR directed against Gpr88 (n = 40) or the negative control miR (n = 40). Then, at T-7, a batch of miR-bearing rats (KD-Gpr88, n = 11; KD-neg, n = 9) were tested for Amph-induced turning behavior and immediately sacrificed. The remaining group of miR-bearing (KD-Gpr88, n = 29; KD-neg, n = 31) rats were tested at T-8 for acute L-DOPA–induced turning behavior. Thereafter, between T-11 and T-14, this batch of rats was chronically (21 days) treated with daily injections of either L-DOPA (KD-Gpr88, n = 18; KD-neg, n = 18) or saline solution (KD-Gpr88, n = 5; KD-neg, n = 5) and serially tested for the development of LID.

The rats were sacrificed by decapitation, and the brains were rapidly removed, frozen in isopentane (−45°C), and kept at −80°C for immunofluorescence and *in situ* hybridization (ISH) experiments.

### Stereotaxic Surgery

The coordinates for stereotaxic surgery were calculated using the Paxinos and Watson rat brain atlas (Paxinos and Watson, 1997). Unilateral injections of 6-OHDA (8 µg in 4.0 µl 0.9% NaCl solution containing 0.02% ascorbic acid) (n = 95) were performed in the left medial forebrain bundle to lesion the nigrostriatal DAergic pathway at the following coordinates (in mm) relative to bregma and the dural surface: antero-posterior (AP) = −1.3, medio-lateral (ML) = +1.3, dorso-ventral (DV) = −8.0, tooth bar at +5. The delivery rate was set at 0.5 µl/min, and at the end of the injection, the needle was left in place for an additional 3 min.

LV-vectors were infused unilaterally in the dorsal striatum on the lesioned side at two dorsal–ventral levels over three sites (2 µl per site, 6 µl total) at the following coordinates (in mm) relative to bregma: (1) AP +1.8, ML +2.8, DV −5.5, −5.0; (2) AP +0.6, ML +2.5, DV −5.5, −5.0; (3) AP +0.6, ML +3.7, DV −5.5, −5.0. The delivery rate was set at 0.33 µl/min, and at the end of the injection, the needle was left in place for an additional 3 min at each site.

### Behavioral Testing for Rotational Behavior

Rotation tests in response to Amph (5 mg/kg, i.p.) were performed in a batch of rats after both 6-OHDA and LV-vector stereotaxic injections by recording the number of turns in an automated rotometer over a 1 h period in time bins of 10 min.

Then, the net turning behavior was calculated for each animal at each 10 min time point by subtracting the number of ipsilateral turns after the 6-OHDA lesion from the number of ipsilateral turns after the lentiviral injection.

The rotational response to an acute injection of L-DOPA (10 mg/kg i.p.) and benserazide (15 mg/kg i.p.) was evaluated after the lentiviral injections. The doses of L-DOPA and benserazide were in the optimal range and proportions reported in the literature to obtain turning behavior (Tayarani-Binazir et al., 2012). Both ipsilateral and contralateral 360° turns were recorded over a 1 h period in time bins of 10 min, and the net rotational asymmetry (contralateral minus ipsilateral turns) was calculated.

### Chronic L-DOPA Treatment

L-DOPA (6 mg/kg, i.p.) plus benserazide (15 mg/kg, i.p.) was injected daily for 21 days to induce a gradual development and a stable degree of LID. The doses of L-DOPA and benserazide were in the optimal range and proportions reported in protocols for developing LID (Cenci et al., 1998; Carta et al., 2006). Control animals received daily injections of saline solution.

### Abnormal Involuntary Movement Rating

LID severity was evaluated by scoring L-DOPA–induced abnormal involuntary movements (AIMs) according to a rat’s dyskinesia scale (Cenci et al., 1998; Cenci and Lundblad, 2007) by an experimenter blind to the type of LV-miR and pharmacological treatment received by the animal.

Rats were placed individually in a transparent plastic cylinder and observed every 20 min for a monitoring period of 1 min, from 20 to 120 min after the injection of L-DOPA or physiological solution.

Ratings of dyskinesia were carried out at days 9, 11, 16, and 18 after the beginning of the chronic L-DOPA treatment. Four subtypes of AIMs were classified: locomotive AIMs (asymmetric locomotion with contralateral turning bias); axial AIMs (twisting of the neck and trunk toward the side contralateral to the lesion); limb AIMs (purposeless “grabbing” movements and/or dystonic posturing of the forelimb contralateral to the lesion); and orolingual AIMs (empty jaw movements and contralateral tongue protrusions). These movements can be clearly discerned from enhanced manifestations of normal motor activities (such as grooming, gnawing, rearing, and sniffing), and they have marked hyperkinetic and/or dystonic features.

Each AIM subtype was scored on a severity scale from 0 to 4 according to the proportion of time/monitoring period during which the AIM was present (0, absent; 1, occasional, i.e. present during less of 50% of the observation time; 2, frequent, i.e. present during more than 50% of the observation time; 3, continuous but interrupted by strong sensory stimuli; 4, continuous, not interrupted by strong sensory stimuli). In case of uncertainty between two consecutive grades of the scale, the corresponding half score was used (i.e. 1.5, 2.5, 3.5). The theoretical maximum sum of AIM scores reached by one rat in one testing session was 96, corresponding to the maximum score/monitoring period (i.e. 16) multiplied by the number of monitoring periods/testing sessions (i.e. 6). Thus, the maximum AIM score that can be attained at the end of the four testing sessions is 96 × 4 = 384.

### Immunofluorescence

Immunofluorescence experiments were performed on 12-µm-thick striatal coronal cryosections spanning the lentivirus-injected area of diffusion. The sections were fixed in 4% paraformaldehyde in phosphate buffered saline (PBS) 1X for 30 min at 4°C, rinsed in PBS 1X, and micro-waved at 700 W for 2.5 min with antigen retrieval (DakoCytomation Dako Glostrup, Denmark). After rinsing in PBS-Triton wash buffer (PBS 1X, 0.2% Triton X-100), sections were blocked with 10% goat serum in PBS1X for 1 h at room temperature. The following primary antibodies were added to a base solution (2% goat serum in PBS1X) and incubated overnight at 4°C: tyrosine hydroxylase (TH) (Millipore MAB318, 1:400), ΔFosB (Abcam AB11959, 1:500), and Neuronal Nuclei (NeuN) (Chemicon MAB 377, 1:400). TH and NeuN immunolabeling was completed using a fluorophore-coupled secondary antibody (Alexa Fluor 647, Invitrogen A21235, 1:1000), while ΔFosB required 3,3′-Diaminobenzidine revelation for best results (BA-2000 secondary antibody, 1:250, and PK6100 kit from Vector Laboratories).

TH and NeuN immunolabeling was completed using a fluorophore-coupled secondary antibody (Alexa Fluor 647, Invitrogen A21235, 1:1000), incubated for 1 h at room temperature. For best results, ΔFosB immunofluorescence required tyramide signal amplification, according to the manufacturer protocol (PerkinElmer, TSA NEL702001KT). The sections were rinsed in PBS-Triton wash buffer three times before being stained with 4’,6-diamidino-2-phenylindole (DAPI) for 10 min. Finally, the sections were rinsed in PBS and mounted using Fluorescent Mounting Medium (Dako Cytomation; Dako, Glostrup, Denmark).

### *In Situ* Hybridization

ISH was performed with antisense digoxygenin-labeled complementary RNA probes designed to recognize *Th*, *Gpr88*, *Gad67*, *Penk*, and *Pdyn* mRNAs. The antisense riboprobes were transcribed from the pGEM^®^-T easy vector as previously described (Ingallinesi et al., 2015).

All plasmids (1 µg/probe) were linearized and used as templates for the synthesis by T7 or SP6 RNA polymerase (Promega, Madison, WI, USA) of the probe labeled with digoxygenin-11-UTP (Roche, Switzerland). The brain sections were processed for ISH as previously described (Ingallinesi et al., 2015).

### Digitization and Semi-Quantitative Analysis

ISH and immunofluorescence slides were digitized using the Axio Scan.Z1 and ZEN software (Zeiss, Oberkochen, Germany). The resulting images were then processed in ImageJ (NIH, Bethesda, MD, USA). As fluorescence and colorimetric staining are not stoichiometrically related to biological content, the signal intensity was not quantified. Instead, a threshold was determined using control slides (secondary antibody alone/sense probe) and applied to all the images from the same experiment. A fixed-size region of interest was then drawn in the transduced areas, where the total signal-positive area was quantified. For each rat, the signal was measured over two to three anteroposterior locations between AP +0.2 mm and +1.8 mm, and averaged. The values from the lesioned/transduced side were then normalized to those obtained in the unaffected hemisphere.

### Statistical Analysis

Rats with 6-OHDA lesion were included for turning behavior statistical analysis when showing individual means >5 full turns per minute in the direction ipsilateral to the lesion with the first Amph challenge. As reviewed by Bjorklund and Dunnet, literature data show that such rotation responses correspond to a permanent reduction in DA content in the striatum of greater than 90% (Bjorklund and Dunnett, 2019). Rats showing less than five full turns per minute (n = 15) were excluded from analysis and discarded from the experimental protocol.

Other inclusion criteria were the complete loss of TH immunoreactivity signal in the striatum or *Th* ISH signal in the *substantia nigra* after the 6-OHDA lesion and detectable EmGFP signals after lentiviral injections. Rats lacking one of these criteria (KD-neg, n = 8; KD-Gpr88, n = 6) were excluded from behavioral and molecular analyses. The number of rats at each experimental step presented in **Figure 1** takes into account these exclusions. Data are presented as mean ± SEM. Statistics were performed using GRAPHPAD PRISM (GraphPad Software Inc., San Diego, CA, USA). One-way or two-way ANOVA followed by *post hoc* Tukey or Bonferroni tests was carried out for statistical analyses as indicated in the results. The distribution of the data from the AIM axial, limb, oral (ALO) locomotor (LOCO) and TOTAL scoring was tested for normality (Kolmogorov–Smirnov test) and then analyzed using a two-way ANOVA followed by Sidak’s multiple comparisons test. The difference between comparisons was considered to be signiﬁcant at P < 0.05.

## Results

### 6-OHDA–Induced Nigrostriatal Lesions and Gpr88 Knock-Down Extension

**Figure 2:**
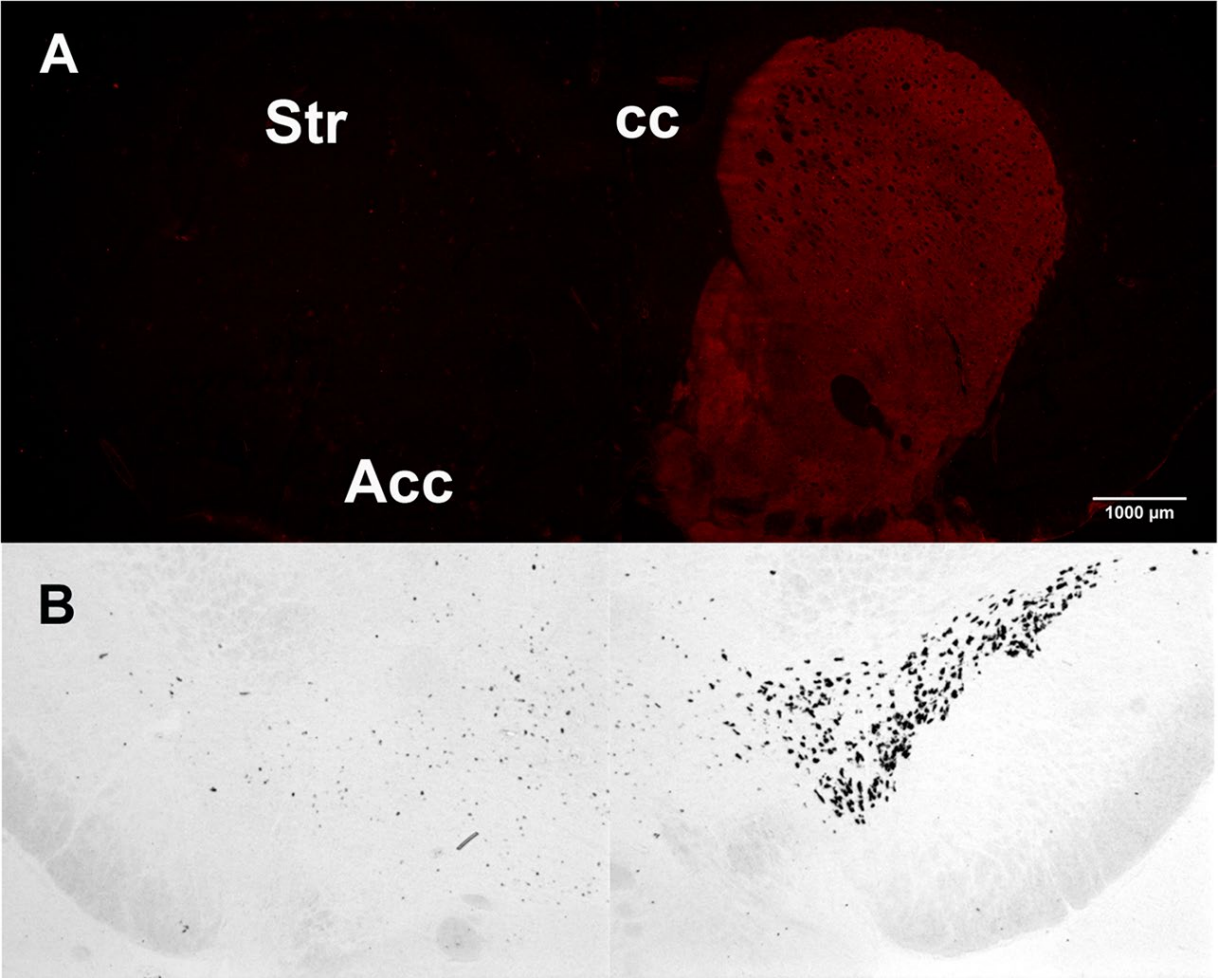
Tyrosine hydroxylase unilateral loss after 6-hydroxydopamine (6-OHDA) lesion. **(A)** Tyrosine hydroxylase immunoreactivity in the 6-OHDA–lesioned (left) and in the intact striatum (right). **(B)** Tyrosine hydroxylase in situ hybridization in the 6-OHDA–lesioned (left) and in the intact substantia nigra (right).

Th, the rate-limiting enzyme in the synthesis of DA and other catecholamines, was used to evaluate the efficacy of the unilateral 6-OHDA injections targeting the nigrostriatal pathway. The 6-OHDA lesion resulted in the complete loss of the specific signal for Th in the side of injection compared to the contralateral intact side, as assessed by immunoreactivity in the striatum and by ISH in the substantia nigra (**Figures 2A, B**).

**Figure 3:**
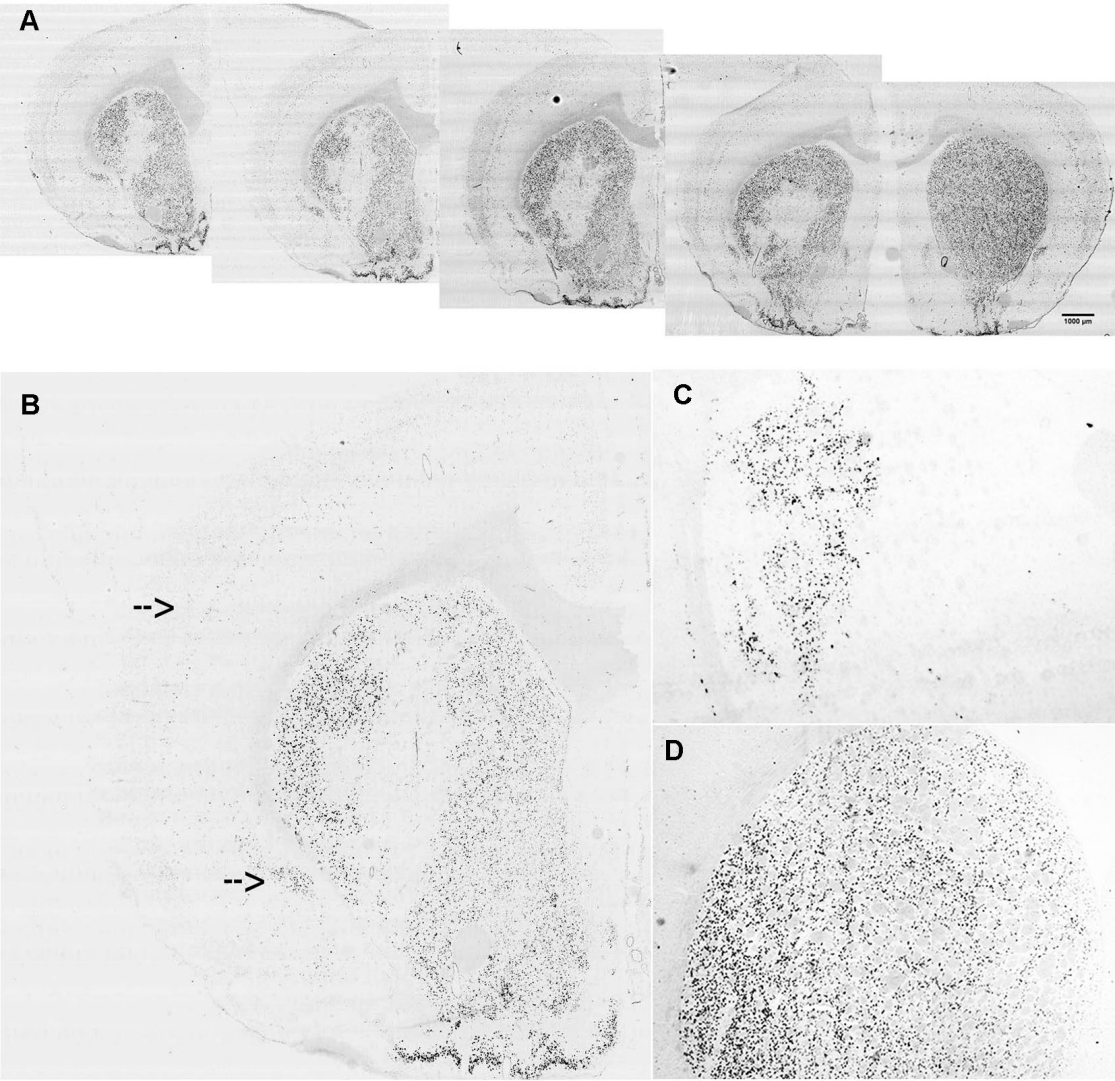
*In situ* hybridization for lentiviral marker and Gpr88 expression. **(A)** Riboprobe against Gpr88 showing the loss of the Gpr88 signal in a series of anteroposterior sections in the region of the left striatum transduced with the specific miR-Gpr88. **(B)** Higher resolution showing the loss of the Gpr88 signal in a section of the left striatum transduced with the specific miR-Gpr88. Arrows indicate Gpr88 expression in the cortex and the piriform cortex. **(C)** Riboprobe against the Emerald green fluorescent protein (EmGFP) marker showing the extension of lentiviral-mediated striatal transduction in a section of the left striatum adjacent to the section reproduced in B. **(D)** Riboprobe against Gpr88 showing the uniform signal of Gpr88 expression in the striatum transduced with the miR-neg.

The lentiviral vectors expressing either the miR-Gpr88 or the miR-neg were injected in the rat dorsal striatum ipsilateral to the 6-OHDA lesion. The diffusion of the lentiviral vector resulted in the localized loss of Gpr88 expression (**Figures 3A, B**) and the presence in the same region of the EmGFP marker (**Figure 3C**) that allowed for ascertaining the efficacy of the transduction. The ISH signal for Gpr88 was, on the contrary, present in the region transduced with the miR-neg (**Figure 3D**).

**Figure 4:**
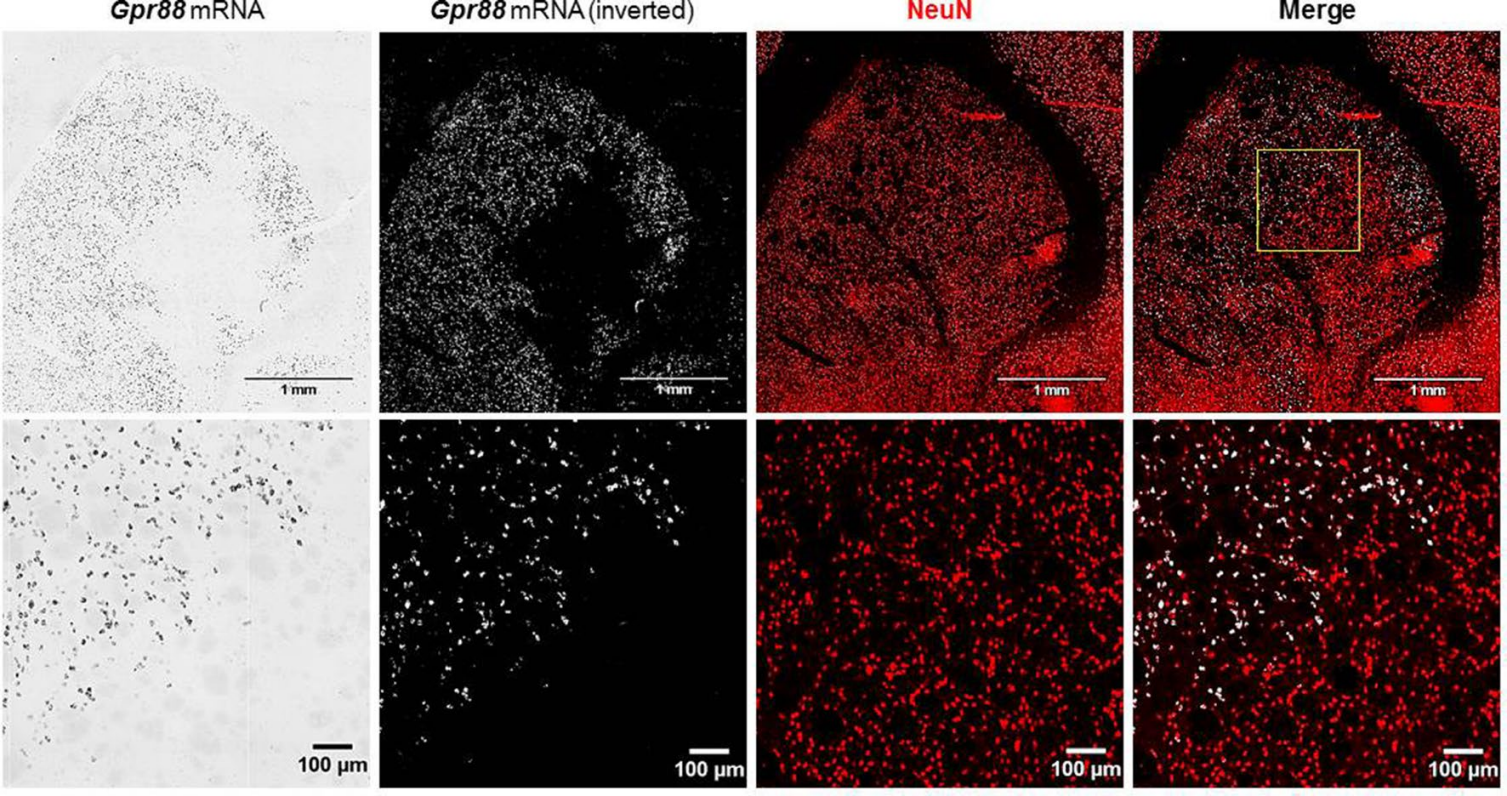
*In situ* hybridization for Gpr88 expression and immunofluorescence for NEUronal Nuclei (NeuN) expression.

The lentiviral-mediated transduction of striatal neurons did not produce any significant neuronal loss, since, as evidenced by the merging of *Gpr88* ISH and NeuN marking, the NeuN signal was uniformly distributed in the striatum without apparent differences between Gpr88-positive and -negative areas (**Figure 4**).

### The KD-Gpr88 Improves Hemiparkisonian Turning Behavior

**Figure 5:**
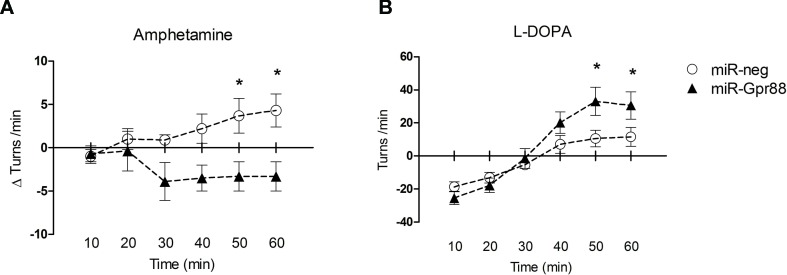
Drug-elicited turning behavior. **(A)** Amphetamine (Amph) (5 mg/kg i.p.)–induced turning behavior: net turning behavior (Δturns) means of the differences calculated for each rat in 10 min interval deducting the number of turns recorded before from the number of turns recorded after lentiviral injection. KD-neg, n = 9; KD-Gpr88, n = 11. Two-way ANOVA: F_1, 108_ = 20.26, p = 0.0001. * p-value <0.05 (Bonferroni post-test). **(B)** L-DOPA (10 mg/kg i.p.) plus benserazide (15 mg/kg i.p.)–induced turning behavior: net rotational asymmetry (contralateral minus ipsilateral turns) after lentiviral vector injections. KD-neg, n = 22; KD-Gpr88, n = 17. Two-way ANOVA: F_5, 270_ = 14.09, p = 0.0001. * p-value <0.05 (Bonferroni post-test).

To ascertain the effects of the KD-Gpr88 on motor activity, we tested the 6-OHDA–lesioned rats, before and after intrastriatal injections of the lentiviral vectors, for Amph-induced turning behavior, which is a highly reliable test for evaluating the extent of DA loss and motor impairment (Iancu et al., 2005; Bjorklund and Dunnett, 2019). Three weeks after the 6-OHDA lesion, Amph elicited a strong turning behavior ipsilateral to the lesioned side (mean 11.7 ± 0.9 turns/min) with no appreciable contralateral turning. The Amph-induced turning behavior was then once again measured in a batch of rats 3 weeks after receiving lentiviral vectors expressing either the miR-neg (KD-neg) or the miR-Gpr88 (KD-Gpr88) in the dorsal region of the DA-depleted striatum. The net turning behavior resulted in positive values in KD-neg rats (n = 9), indicating that the effect of the 6-OHDA lesion increased over time. On the contrary, the net turning behavior resulted in negative values in the KD-Gpr88 rats (n = 11), showing that the Gpr88 inactivation partially reduced the motor imbalance induced by 6-OHDA lesions (**Figure 5A**).

Four weeks after lentiviral vector injections, turning behavior following an acute L-DOPA (10 mg/kg i.p.) plus benserazide (15 mg/kg i.p.) challenge was assessed in another batch of 6-OHDA–lesioned and lentiviral transduced rats that were not previously tested with a second Amph treatment. In the 6-OHDA–lesioned rats, initial turning toward the lesioned site was observed, which then switched to the contralateral side after half an hour, an effect of acute L-DOPA treatment that has been reported in the literature (Mura et al., 2002). The contralateral turning was increased in the KD-Gpr88 as compared to KD-neg rats, indicating that the Gpr88 inactivation enhances the effects of L-DOPA in the 6-OHDA–lesioned striatum (**Figure 5B**).

### KD-Gpr88 and L-DOPA–Induced Dyskinesia

Two 6-OHDA–lesioned groups of rats, bearing either the KD-neg or the KD-Gpr88, were chronically treated for 21 days with L-DOPA (6 mg/kg) plus benserazide (15 mg/kg), while two corresponding groups of animals received an equal volume of saline solution. The 6-OHDA–lesioned rats chronically treated with saline (KD-neg, n = 5; KD-Gpr88, n = 5) did not develop LID. On the contrary and as expected according to the literature (Cenci et al., 1998), comprehensively over 50% (n = 20) of the 6-OHDA–lesioned rats chronically treated with L-DOPA developed LID, both in the KD-neg (n = 8 out of 18) and in the KD-Gpr88 (n = 12 out of 18) group. The distribution of the data from the AIM scoring (ALO, LOCO, and TOTAL scores) was verified and passed the normality testing using the Kolmogorov–Smirnov test.

The total AIM score in the LID-positive animals was of about 55% of the maximal theoretical score of 384 (Cenci and Lundblad, 2007) (see *Materials and Methods*) and was not different between KD-neg (222.7 ± 27.9; n = 8) and KD-Gpr88 (206.4 ± 17.3; n = 12).

**Figure 6:**
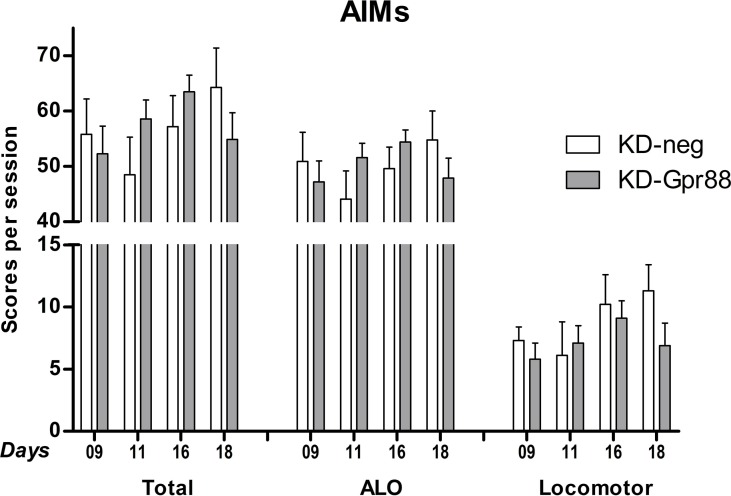
L-DOPA–induced dyskinesia (LID): abnormal involuntary movement (AIM) scores. AIMs were scored at each time point (9, 11, 16, and 18 days of L-DOPA treatment) and calculated either as the total sum or separating the sum of the ALO (axial, limb, oral) scores from the locomotor scores. The data were analyzed using a two-way ANOVA followed by Sidak’s multiple comparisons test. The analysis did not reveal a significant effect of the miR treatment (F_1, 57_ = 0.06, p = 0.81), time (F_3, 57_ = 1.02, p = 0.39), or their interaction (F_3, 57_ = 1.53, p = 0.22).

Also, the AIM score at each time point (9, 11, 16, and 18 days of L-DOPA treatment) calculated either as a whole or separating the ALO (axial, limb, oral) scores from the locomotor scores was not different between the two experimental groups (**Figure 6**). Thus, these results show that, notwithstanding the acute L-DOPA–elicited increase in contralateral turning behavior, the KD-Gpr88 does not worsen the severity of LID following a chronic L-DOPA treatment.

### The KD-Gpr88 Normalizes the 6-OHDA–Induced *Gad67* and *Penk* Overexpression and Prevents LID-Associated *Pdyn* Upregulation

The chronic L-DOPA treatment associated with the development of LID increases the expression of *Gad67*, *Penk*, and *Pdyn*, with this latter being positively correlated with AIM scores (Cenci et al., 1998). Moreover, *Pdyn* upregulation associated with LID is promoted by ΔFosB (Andersson et al., 1999). Thus, we assessed the expression of *Gad67*, *Penk*, and *Pdyn* in the dorsal striatum of 6-OHDA–lesioned KD-neg and KD-Gpr88 rats that developed LID after 21 days’ treatment with L-DOPA and their corresponding controls treated for 21 days with a saline solution.

**Figure 7:**
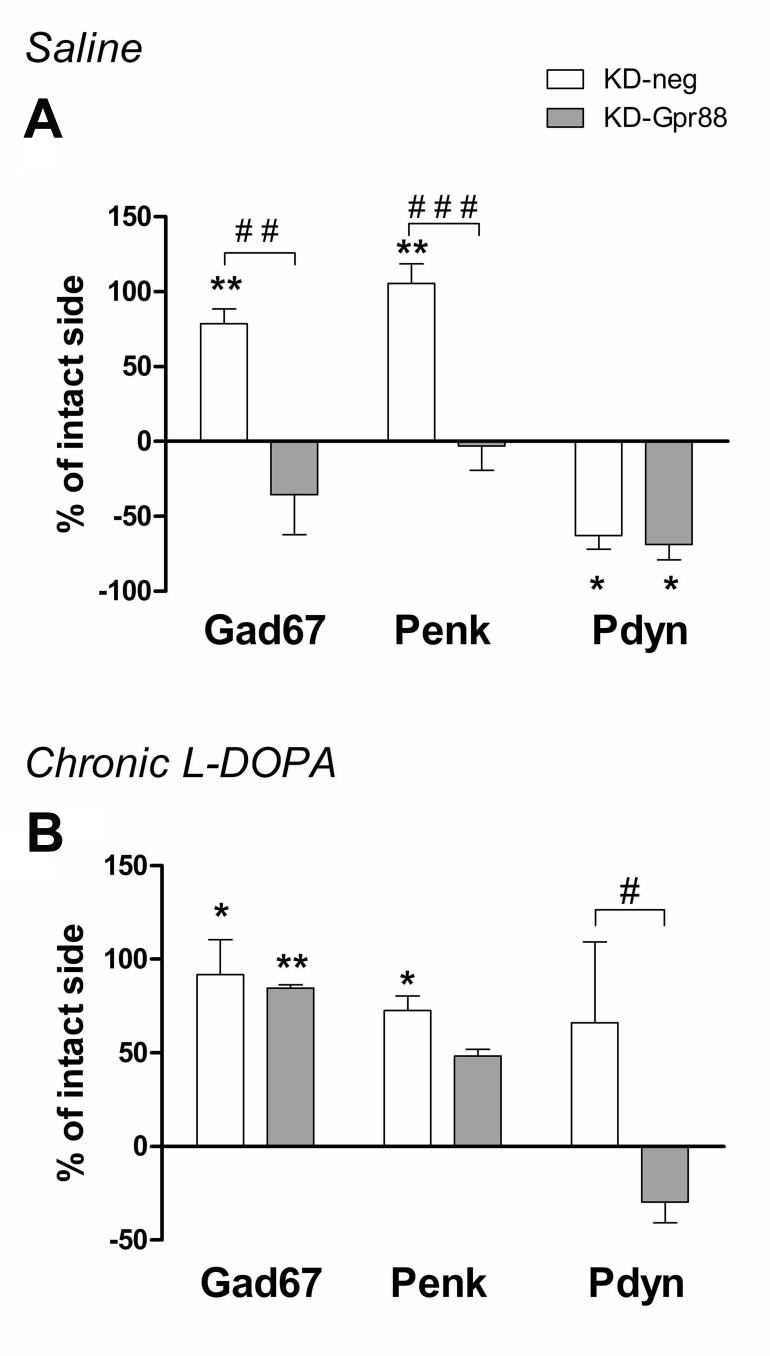
*In situ* hybridization for striatal markers. *Gad67*, *Penk*, *Pdyn* mRNA expression. Evaluation of medium spiny neuron markers in the dorsal DA-deprived striatum transduced with a miR-neg or miR-Gpr88 expressed as percentage of change compared to the contralateral intact side in chronic saline control rats and chronic L-DOPA–treated rats developing LID. **(A)** Chronic saline: KD-neg, n = 4; KD-Gpr88, n = 3. **(B)** Chronic L-DOPA: KD-neg, n = 3; KD-Gpr88, n = 4. One-way ANOVA: control side v/s KD-neg v/s KD-Gpr88 followed by Tukey multiple comparison test. * 6-OHDA–lesioned v/s intact side—P: * < 0.05, ** < 0.01. # KD-neg v/s KD-Gpr88—P: # < 0.05, ## < 0.01, ### < 0.005.

As expected according to the literature (Cenci et al., 1998), the 6-OHDA lesion increased *Gad67* and *Penk* and decreased *Pdyn* expression in KD-neg rats. However, the KD-Gpr88 reversed to baseline levels both *Gad67* and *Penk* but not *Pdyn* expression (**Figure 7A**). LID is associated with *Gad67*, *Penk*, and *Pdyn* overexpression. Accordingly, in LID-displaying KD-neg rats, the expression of *Gad67*, *Penk*, and *Pdyn* was concomitantly increased. However, in the KD-Gpr88 rats with LID, while *Gad67* and *Penk* were upregulated to the same extent as in their KD-neg counterparts, *Pdyn* expression remained inferior to the baseline and significantly different from *Pdyn* expression in the KD-neg rats (**Figure 7B**), indicating that the Gpr88 inactivation prevents the *Pdyn* upregulation that is associated with the development of LID (Cenci et al., 1998).

### Hyperactivation of ΔFosb Following Gpr88 Inactivation

LID-associated *Pdyn* upregulation, which is positively correlated with AIM scores, is promoted by ΔFosB activation (Cenci et al., 1998). Actually, the L-DOPA chronic treatment after 6-OHDA lesions has been shown to increase the levels of ΔFosB, a member of the Fos family of transcription factors that is induced by repetitive stimulation and that accumulates in a stable form in the D1-expressing MSN of the direct pathway (Nestler, 2015). Since the accumulation of ΔFosB is also correlated with the severity of LID (Pavon et al., 2006; Engeln et al., 2016), we evaluated the effects of the Gpr88 inactivation on ΔFosB expression.

**Figure 8:**
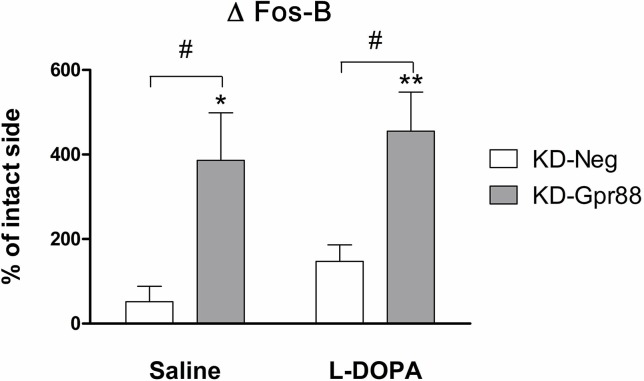
ΔFosB protein expression. Immunofluorescence analysis of ΔFosB activation after chronic saline or L-DOPA treatment in the lentiviral vector transduced dorsal striatum of 6-OHDA–lesioned rats developing LID. Chronic saline: KD-neg, n = 4; KD-Gpr88, n = 4. Chronic L-DOPA: KD-neg, n = 3; KD-Gpr88, n = 4. One-way ANOVA: Control side v/s KD-neg v/s KD-Gpr88 followed by Tukey multiple comparison test. * 6-OHDA–lesioned v/s intact side—P: * < 0.05; ** < 0.01. # KD-neg v/s KD-Gpr88—P: # < 0.05.

Strikingly, in the dorsal striatum of KD-Gpr88 rats, ΔFosB was strongly increased both in saline (n = 4) and in chronic L-DOPA (n = 4) treated rats as compared to KD-neg (**Figure 8**). Thus, the Gpr88 inactivation powerfully induced ΔFosB expression in the DA-deafferented striatum but was not associated with any further increase of ΔFosB following chronic L-DOPA treatment. Moreover, since the L-DOPA–induced *Pdyn* upregulation is inhibited, the increased accumulation of ΔFosB appeared to be unable to worsen LID in L-DOPA–treated KD-Gpr88 rats.

## Discussion

DA modulates MSN output by facilitating the activity of the D1-regulated striato-nigral pathway and inhibiting the activity of D2-regulated striato-pallidal pathway (Gerfen and Wilson, 1996). The imbalance between these striatal output pathways following unilateral DA loss can be evaluated measuring the turning behavior that results from the difference between the intact and lesioned striatum in the response to activation of the DA receptors. Turning behavior can thus be elicited by acute challenges with drugs such as Amph that, by increasing the release of DA in the intact striatum, provokes turning toward the lesioned side, or the DA precursor L-DOPA that, by promoting DA release in the DA-hypersensitive 6-OHDA–lesioned striatum, produces turning toward the intact side (Schwarting and Huston, 1996). We have found that the KD-Gpr88 in the dorsal DA-deafferented striatum produced a significant effect on motor activity in such a model of PD by attenuating the ipsilateral Amph- and enhancing the contralateral L-DOPA–induced turning behavior. This effect is consistent with an inhibitory role of Gpr88 on DA-dependent MSN activity, since Gpr88 KO mice display DA hypersensitivity, as shown by increased locomotion in basal conditions and in response to DA receptor stimulation with direct and indirect agonists (Logue et al., 2009; Quintana et al., 2012).

Using a threshold method for ISH analysis, we ascertained that, as already reported in the literature (Cenci et al., 1998), the DA loss following 6-OHDA lesions leads to hyperactivation of MSN with an increase in the expression of *Gad67*, coding for the rate-limiting enzyme in the synthesis of GABA, and also results in the decreased expression of *Pdyn* co-transmitter in the striato-nigral pathway and in the increased expression of *Penk* co-transmitter in the D2 striato-pallidal pathway. These modifications are the result of plastic changes triggered in the striatum by the loss of DA fibers, and reflect an imbalance in the activity of striatum output pathways, giving rise to motor deficits. The KD-Gpr88 normalized the expression of *Gad67* and *Penk*. However, it was unable to modify the decrease in *Pdyn* expression induced by the DA loss. Thus, in a well-established, unilateral, 6-OHDA lesion model of PD, the KD-Gpr88 appears to reduce the imbalance in motor responses to DA receptor stimulation essentially by normalizing the activity of the indirect inhibitory striato-pallidal pathway. This suggestion that KD-Gpr88 preferentially affects MSNs of the indirect pathway is consistent with the receptor’s relative enrichment in this neuronal type (Massart et al., 2009) and the hypersensitivity to D2 agonists that has been consistently reported in Gpr88 KO mice (Logue et al., 2009; Quintana et al., 2012). As GPR88 is also known to modulate enkephalin delta opioid receptor signaling (Meirsman et al., 2016), the reduction in Penk expression observed following KD-Gpr88 may partly result from a potentiation of neurotransmission at delta receptors. However, the KD-Gpr88, by not modifying the 6-OHDA–induced *Pdyn* downregulation, appears to not markedly impact the direct striato-nigral pathway that may remain hypersensitive upon D1 receptor stimulation. This may explain the lack of effects of Gpr88 long-term inactivation alone on the development of LID in contrast to a chronic treatment with L-DOPA. Actually, L-DOPA replacement therapy for the treatment of PD facilitates motor function by enhancing the activity of the D1-regulated striato-nigral pathway, an effect that, in the long term, leads to the development of LID (Santini et al., 2009). In this study, we found that the KD-Gpr88, even if it induced an increase of contralateral turning behavior after an acute challenge with L-DOPA, did not exacerbate the intensity of AIMs that are associated with the chronic L-DOPA–induced hyperactivation of the direct pathway (Santini et al., 2009).

Moreover, the development of LID is associated with an upregulation of *Gad67* and *Penk*, which is more pronounced in term of signal intensity than the upregulation induced by the DA deafferentiation, an upregulation of *Pdyn*, and the induction of ΔFosB in the direct pathway that is linked to the severity of LID (Pavon et al., 2006; Engeln et al., 2016). However, only *Gad67* and *Penk* were upregulated to the same extent in both KD-neg and KD-Gpr88 rats with LID, while the increase of *Pdyn* in KD-neg was not paralleled in KD-Gpr88 rats, where it remained below baseline. Furthermore, ΔFosB was significantly increased by about threefold both in saline- and L-DOPA–treated KD-Gpr88 as compared to KD-neg rats. Nevertheless, the sharp increase of ΔFosB in saline-treated KD-Gpr88 rats was not associated with the development of AIMs. Accordingly, it has been reported that high levels of ΔFosB expression obtained by viral vector transduction in the DA-denervated striatum are not *per se* associated with dyskinesia. However, the overexpression of ΔFosB results in the abrupt appearance of high-score AIMs immediately after an acute L-DOPA challenge, which are significantly increased compared to the AIM scores displayed after chronic L-DOPA treatment (Cao et al., 2010). This is not the case for the sharp increase in ΔFosB induced by the KD-Gpr88, which is not associated with the development of dyskinesia and with a significant increase in AIM scores after chronic L-DOPA. However, similarly to the KD-Gpr88, the overexpression of ΔFosB results in hypersensitivity to acute L-DOPA and increased contralateral turning (Cao et al., 2010). Thus, the lack of dyskinesia development and the lack of aggravation of AIMs after the dorso-striatal inactivation of Gpr88 suggest that, while the KD-Gpr88 may act by hyperactivating the D1 direct pathway through the induction of ΔFosB, this effect is not directly coupled to dyskinetic effects even during a chronic L-DOPA treatment. Indeed, ΔFosB upregulates *Pdyn* (McClung et al., 2004), which is linked to both development and severity of LID (Andersson et al., 1999; Andersson et al., 2003). In this study, following chronic L-DOPA treatment, we observed a significant increase of *Pdyn* in KD-neg and a stable level of *Pdyn* in the KD-Gpr88 group, this latter group being also characterized by a striking increase in ΔFosB protein levels. Thus, this result suggests that the KD-Gpr88 may also affect the D1 direct pathway by inhibiting the upregulation of Pdyn following chronic L-DOPA and, therefore, that LID may not depend only on upregulated ΔFosB/*Pdyn* in the D1 direct pathway but also on a potential involvement of other cellular mechanisms in different neurotransmission systems such as the serotonergic system (De Deurwaerdère et al., 2017).

Taken together, our results indicate that the KD-Gpr88 may act on both the hyperactive indirect and hypoactive direct pathways following the 6-OHDA lesion and, by reducing the imbalance between them, may result in an antiparkinsonian-like effect not associated in the long term with the development of dyskinesia. However, the development of specific pharmacological antagonists will be crucial for further establishing whether GPR88 receptors genuinely represent an alternative target for the treatment of PD—alone or in association with other classes of agents—with a lower propensity to provoke motor side effects. Moreover, using specific D1 or D2 ligands in PD models combined with the KD-Gpr88 will be instrumental for parsing the specific contribution of Gpr88 to the direct and indirect pathways. However, to disentangle the relative contribution of each pathway in the effect of the Gpr88 KD would also require the utilization of lentiviral vectors with specific promoters. This will allow for dissecting more precisely the role of Gpr88 and for developing gene therapy tools that may offer alternative and possibly more efficient solutions than pharmacological interventions for treating the motor dysfunction of PD while avoiding the eventual emergence of dyskinesia.

## Data AvailaBility Statement

The datasets generated for this study are available on request to the corresponding author.

## Ethics Statement

The animal study was reviewed and approved by “Ministère de la Recherche” (APAFIS#3669-2016011817516297 v6).

## Author Contributions

MI, MM, CM, and RM conceived and organized the research project. MI, BG, JP, NF, ADT, and RM planned and executed the experimental work. MI, BG, and RM designed and executed the statistical analysis. MI wrote the first draft of the manuscript. MI, BG, NF, ADT, MM, and CM reviewed and provided scientific input to the manuscript. RM wrote the article.

## Conflict of Interest

The authors declare that the research was conducted in the absence of any commercial or financial relationships that could be construed as a potential conflict of interest.
